# Leucine Promotes Proliferation and Differentiation of Primary Preterm Rat Satellite Cells in Part through mTORC1 Signaling Pathway

**DOI:** 10.3390/nu7053387

**Published:** 2015-05-08

**Authors:** Jie-Min Dai, Mu-Xue Yu, Zhen-Yu Shen, Chu-Yi Guo, Si-Qi Zhuang, Xiao-Shan Qiu

**Affiliations:** Department of Pediatrics, The First Affiliated Hospital, Sun Yat-sen University, Guangzhou 510080, China

**Keywords:** leucine, preterm, satellite cells, proliferation, differentiation, mTOR, MyoD, myogenin

## Abstract

Signaling through the mammalian target of rapamycin (mTOR) in response to leucine modulates many cellular and developmental processes. However, in the context of satellite cell proliferation and differentiation, the role of leucine and mTORC1 is less known. This study investigates the role of leucine in the process of proliferation and differentiation of primary preterm rat satellite cells, and the relationship with mammalian target of rapamycin complex 1 (mTORC1) activation. Dissociation of primary satellite cells occurred with type I collagenase and trypsin, and purification, via different speed adherence methods. Satellite cells with positive expression of Desmin were treated with leucine and rapamycin. We observed that leucine promoted proliferation and differentiation of primary satellite cells and increased the phosphorylation of mTOR. Rapamycin inhibited proliferation and differentiation, as well as decreased the phosphorylation level of mTOR. Furthermore, leucine increased the expression of MyoD and myogenin while the protein level of MyoD decreased due to rapamycin. However, myogenin expressed no affect by rapamycin. In conclusion, leucine may up-regulate the activation of mTORC1 to promote proliferation and differentiation of primary preterm rat satellite cells. We have shown that leucine promoted the differentiation of myotubes in part through the mTORC1-MyoD signal pathway.

## 1. Introduction

Limited energy reserves are common in preterm infants at birth. In order to prevent a catabolic state, adequate provision of calories and protein is needed to match intrauterine accretion rate soon after birth. The use of aggressive amino acids intake is associated with increased protein accretion and decreased extrauterine growth restriction (EUGR). EUGR can have an impact on an infant’s later neurodevelopment, growth outcomes and metabolic disorders [1,2,3]. 

Higher protein intake (≥3.0 g/kg/day but <4.0 g/kg/day) from formula accelerates weight gain [4]. Based on increased nitrogen accretion rates, this most likely suggests an increase in lean body mass. We have proven that high-protein intervention significantly alters the body composition of low birth weight rat offspring, with an increased percentage of lean mass and decreased percentage of fat mass [5]. The mechanism related to high-protein intervention deserves further study.

Leucine is one of the essential amino acids recommended in preterm formula at the highest level, about 252–362 mg/100 Kcal [6]. Indeed, leucine is the most effective single amino acid to activate protein synthesis in skeletal muscle [7]. There is now strong evidence that leucine enhances mammalian target of rapamycin complex 1 (mTORC1) signaling, as well as represses proteasomal degradation to increase skeletal muscle protein synthesis [8,9].

Mammalian target of rapamycin (mTOR) is an atypical serine/threonine protein kinase that functions as a master regulator of cell growth, proliferation, and various types of cellular differentiation [10]. There exist two distinct multi protein complexes, namely mTORC1 and mammalian target of rapamycin complex 2 (mTORC2), that mediate rapamycin-sensitive and rapamycin-insensitive signaling of mTOR respectively [11]. Raptor-associated mTORC1 assembles a signaling network that receives signals arising from growth factors, nutrients, and cellular energy metabolism. One of the best-characterized downstream targets of mTORC1 is ribosomal protein S6 kinase, polypeptide 1 (S6K1), the ribosomal S6 kinase that regulates protein synthesis at the translational initiation level [12]. Leucine is well-known regulator of the protein kinase mTOR when it is part of the mTORC1 complex [13]. Furthermore, several studies have focused on the role of mTORC1 in the regulation of protein synthesis in muscle in response to amino acids [14].

Skeletal muscle is the largest component of lean body mass in humans. It is essential for lifelong metabolic health [15,16]. In the process of muscle generation, satellite cells are the major muscle stem cells, which are located in the periphery of muscle fibers between the lamina and the plasma membrane [17]. The process of generating muscle-myogenesis-involves several steps including proliferation, migration, and fusion of satellite cells either with an existing fiber or with other satellite cells to form a new muscle fiber [18,19]. A family of muscle-specific transcription factors called myogenic regulatory factors (MRFs) mainly regulates this process. The MRF subfamily consists of myf5, MyoD, myogenin and MRF4, express in sequence during differentiation [19]. Among the four MRFs, MyoD is the best-characterized one and has been demonstrated to promote the withdrawal of myoblasts from cell cycle and the induction of myoblast differentiation [20]. Moreover, deficiency of MyoD in primary satellite cells of mice leads to a defect of differentiation [21,22]. Myogenin mediates the differentiation process of myotube formation and the contractile protein synthesis [23]. Indeed, myogenin has a unique function in the transition from a determined myoblast to a fully differentiated myotube [24].

It has been reported that reduction of MyoD induced by rapamycin treatment inhibits myogenic differentiation in C2C12 cells [25,26]. To date, there are no studies in primary preterm rat satellite cells. Furthermore, there is little exploration about the effects of leucine and mTORC1 on satellite cell proliferation and differentiation. This study investigates the role of leucine in the process of the proliferation and differentiation of primary preterm rat satellite cells, and their relationship with mTORC1 signal pathways. 

## 2. Experimental Section

### 2.1. Animals

Specific pathogen-free Sprague Dawley rats weighted 220–250 g at 3 months of age. The rats were from the Animal Experiment Center of First Affiliated Hospital of Sun Yat-sen University and placed at room temperature (25 °C) with a 12-h-light/12-h-dark cycle. We arranged male rats to mate with female rats at a ratio of 1:1. 

The day of sperm cell detection in female rats is day 1 of gestation. These preterm rats were born by caesarean section on day 18 of gestation. The Ethics Committee of the First Affiliated Hospital of Sun Yat-sen University approved the experiment. 

### 2.2. Reagents

We obtained Antibodies against mTOR (#2983) and mTOR Phospho-Ser2448 (#5536) from Cell Signaling Technology. Abcam provided Antibodies against S6K1 (ab32359) and S6K1 Phospho-thr389 (ab2571). Antibodies against MyoD (#554130) and Myogenin (#556358) came from BD Biosciences. Anti-desmin polyclonal antibody was purchased from Thermo Fisher Scientific; and HRP-conjugated anti-mouse and anti-rabbit IgG antibodies were supplied by EarthOx and Abmart, respectively. We purchased DMEM/Nutrient Mixture F-12 Ham (D9785) and Leucine from Sigma-Aldrich, as well as Type I Collagenase and Trypsin. Fetal bovine serum (FBS) was purchased from Gibco Life Technologies. Dojindo Molecular Technologies provided Cell Counting Kit-8 (CCK-8). The Rapamycin and Basic Fibroblast Growth Factor (b-FGF) were from BBI Solutions. 

### 2.3. Primary Culture of Preterm Rat Satellite Cells

Primary satellite cells came from limb muscle of preterm newborn rats. We incubated finely chopped muscle in 0.1% type I collagenase solution. After 30 min of digestion, cells were centrifuged (10 min, 180 g) and the supernatant removed. We then added 0.25% trypsin solution. After 15 min of incubation, we added Dulbecco’s Modified Eagle’s Medium/Nutrient F-12 Ham (DMEM/F12ham) with 20% FBS to terminate digestion. In succession, cells passed through a 70-μM cell strainer, a 40-μM cell strainer, and centrifuged again (10 min, 180 g). The pellet was then suspended with growth medium which was supplemented with DMEM/F12ham, 20% FBS, 10%HS and 5ng/ml bFGF. We plated suspended cells on collagen-coated dishes. Then pre-plating was complete on petri dishes. Two hours later, we transferred the supernatant containing nonadherent cells into new collagen-coated dishes. We changed the medium two days later, then subsequently changed each day. The cells were treated with trypsin upon reach of approximately 50%–60% confluence. One hour of pre-plating on Petri dishes was completed before plating the cells on collagen-coated dishes. These two steps of pre-plating reached purity beyond 90% of satellite cells. 

### 2.4. Differentiation Culture

To initiate differentiation, cells were cultured when reaching approximately 80% confluence in DMEM/F12ham devoid in leucine with 2% horse serum for 3 days, with different concentrations of leucine. We changed the medium each day. We observed and photographed myotubes under an inverted fluorescence microscope.

### 2.5. Immunocytochemical

To determine the percentage of cells expressing a specific muscle cell marker, we stained the cells with the desmin antibody. Cells cultured on collagen-coated glass coverslips were fixed in 4% paraformaldehyde for 10 min at room temperature and then permeabilized with phosphate buffered saline (PBS) containing 0.1% Triton X-100 for 15 min. After blocking with 1% BSA solution for 30 min, the cells were incubated in the diluted primary antibody against desmin (1:200), overnight, in a humidified chamber at 4 °C. After three washings with PBS, biotinylated goat anti rabbit secondary antibogy IgG was added. We allowed it to incubate for 30 min at room temperature. Then, after again washing with PBS three times, we added streptavidin/peroxidase immunohistochemical reagent to soak for 20 min at room temperature. To visualize immunoreactivity, coverslips were incubated in 3,3’-diaminobenzidine (DAB) peroxidase substrate for 20 to 30 s, and then washed in double distilled H_2_O, counterstained, and dehydrated. We used an optical microscope to take images.

### 2.6. Cell Proliferation Rate Assay

Cell suspensions (100 μL) were seeded on 96-well plates (1000 cells/well) and incubated for 24 h at 37 °C in 5% CO_2_. Subsequently, cells were starved for serum in DMEM/F12ham free-serum for 4 h. Following starvation, we cultured cells in DMEM/F12ham devoid of leucine without serum for another 4h with different concentrations of leucine. Later, each well received 10-μL CCK-8 solution, and the cells were incubated for an additional 3 h. The colorimetric was measured at 450 nm with a microplate reader to obtain an optical density (OD) value. 

### 2.7. Western Blot

After all treatments, primary satellite cells were washed with PBS, and then lysed in a lysis buffer that contained a protease inhibitor, phenylmethyl sulfonyl fluoride. We measured protein concentrations by using the bicinchoninic acid method, according to the manufacturer’s instructions. We separated equal amounts (30-μg) of protein from each sample by SDS/PAGE and then transferred in to a polyvinylidene difluoride membrane. After incubated in blocking buffer for one hour at room temperature, the membranes were incubated overnight at four degrees celsius with primary antibodies against mTOR (1:1000), mTOR Phospho-Ser2448 (1:1000), S6K1 (1:2000), S6K1 Phospho-thr389 (1:500), MyoD (1:500), Myogenin (1:2000) and GAPDH (1:10000), followed by incubation with appropriate horseradish peroxidase-linked secondary anti-bodies. Signals became obvious after using enhanced chemiluminescence and then quantitated using Image J. The ratio of the expression of target proteins was determined after normalizing the individual GADPH levels. We repeated each experiment three times.

### 2.8. Statistical Analysis

All data were expressed as the means ± SD. We used ANOVA to assess statistical differences between mean values, followed by the least significant difference *t* test to determine statistical significances. All statistical analyses were performed using SPSS 13.0. Significance is defined at the 0.05 level.

## 3. Results

### 3.1. Culture and Identification of Primary Preterm Rat Satellite Cells

**Figure 1:**
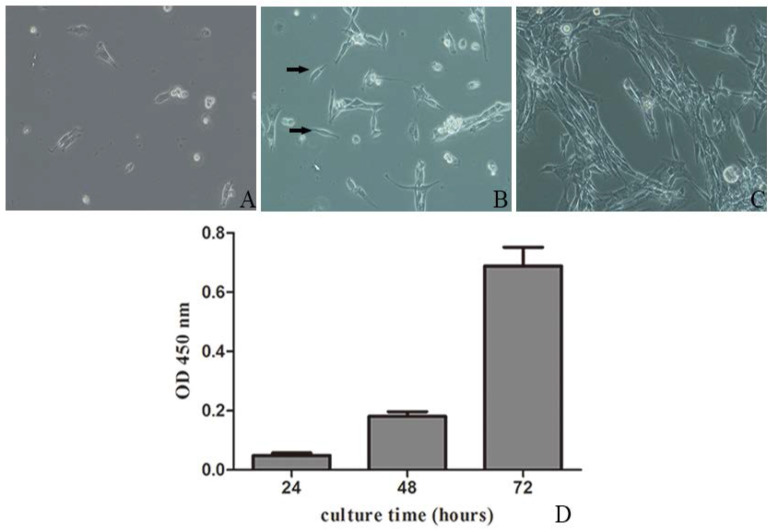
Micrographs of primary preterm rat satellite cells. (**A**) 24 h after isolation; (**B**) 48 h after isolation (arrows: typical skeletal satellite cell, with short spindle shape and high refractive index); (**C**) 72 h after isolation (×200); (**D**) Cell proliferation analysis. Cells were seeded on 96-well plates, and cells proliferation was assessed using Cell Counting Kit-8 (CCK-8) assay every 24 h. Data are shown as the mean ± SD of three independent experiments.

Primary preterm rat satellite cells, freshly isolated and purified were round, small, and refracted under phase contrast microscope. After 24 h, some satellite cells began to adhere to the wall and extend gradually, with a short spindle shape and high refractive index (Figure 1A). Satellite cells were fully adherent and extended by 48 h (Figure 1B). After 72 h, satellite cells were proliferating obviously, and arranged in parallel (Figure 1C). As shown in Figure 1D, the extent of proliferation of the cells was obvious. When the cells reached 50%–60% confluences, they should be trypsinized to subculture. The passage cell began to adhere to the wall within 1 h after plating, with morphology similar to primary cells. 

**Figure 2:**
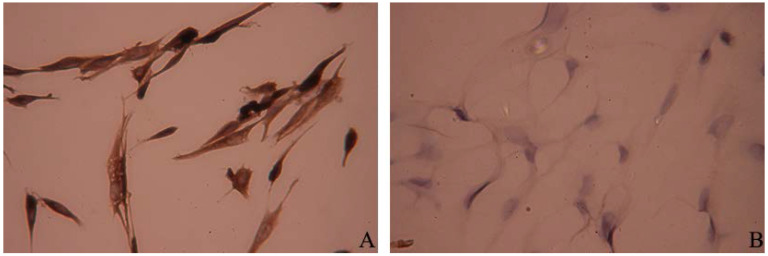
Immunocytochemistry staining of primary preterm rat satellite cells. (**A**) Primary satellite cells immunochemistry stained by desmin antibody; (**B**) Fibroblasts immunochemistry stained by desmin antibody as negative control (×400).

Immunocytochemistry analysis showed that cytoplasm of primary satellite cells were stained in brown-yellow color, indicating that desmin expressed in the cytoplasm of the cells (Figure 2A). Fibroblasts immunochemistry stained with antibody to desmin showed negative reaction (Figure 2B).

### 3.2. Leucine Promotes Primary Satellite Cells Proliferation Through mTORC1 Pathway

**Figure 3:**
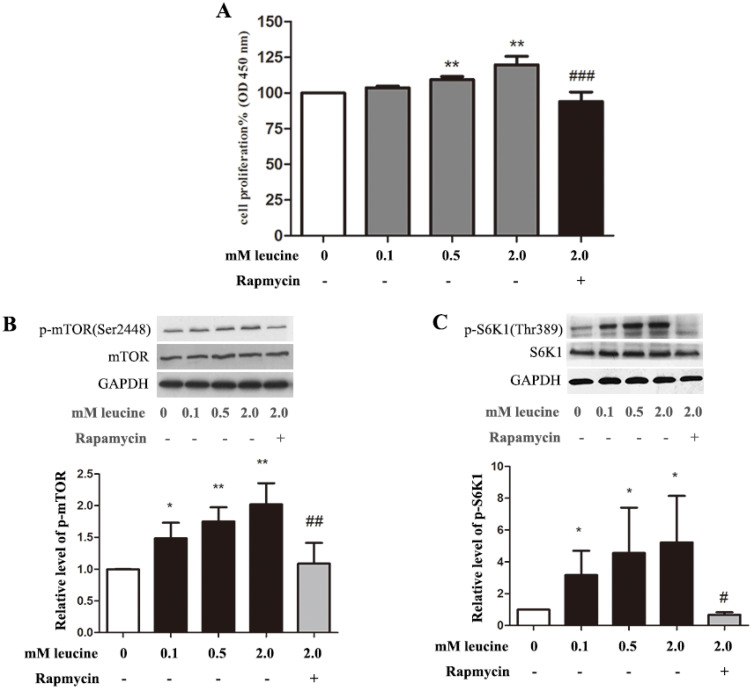
Leucine promotes primary satellite cell proliferation through the mammalian target of rapamycin complex 1 (mTORC1) pathway. (**A**) Proliferation activity of primary satellite cells was enhanced by leucine in a dose-dependent manner. When indicated by “+”, cells were treated with 50 nM rapamycin. ** *p <* 0.01 *vs.* control (0 mM leucine). ^###^ *p <* 0.001 *vs.* 2.0 mM leucine. Data are shown as the mean ± SD of six independent experiments; (**B**) The expression levels of mTOR, phospho-mTOR and GAPDH. Primary satellite cells starved for serum in DMEM/F12ham free-serum for 4 h. After starvation, cells were cultured in DMEM/F12ham devoid of leucine, without serum for another hour, with different concentrations of leucine, followed by Western analysis. The values were adjusted to total mTOR intensity and then normalized to expression from the control group (0 mM leucine); (**C**) The expression levels of S6K1, phospho-S6K1 and GAPDH. Cells were treated the same as decribed in (**B**); The values were adjusted to total S6K1 intensity and then normalized to expression from the control group (0 mM leucine); (**B**,**C**) data are shown as the mean ± SD of three independent experiments and representative images are shown. * *p <* 0.05, ** *p <* 0.01 *vs.* control (0 mM leucine). ^#^ *p <* 0.05, ^##^ *P <* 0.01 *vs.* 2.0 mM leucine.

Proliferation assay in primary preterm rat satellite cells was complete by treating cells with leucine of different concentrations. CCK-8 assays indicated that leucine enhanced primary satellite cell proliferation was in a dose-dependent manner, while rapamycin inhibited cell proliferation (Figure 3A). The results of western blot showed that leucine activated phosphorylation of mTOR and S6K1 also reacted in a dose-dependent manner in proliferating cell, and the proliferation promotion stopped after mTOR pathway blockage by rapamycin (Figure 3B,C).

### 3.3. Leucine Promotes Differentiation of Primary Preterm Rat Satellite Cells

**Figure 4:**
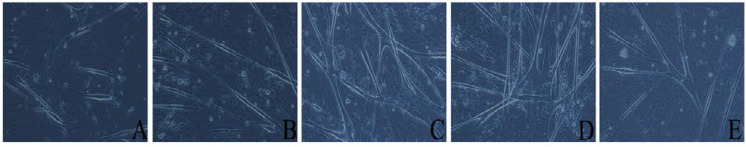
Views of myotube raised in differentiation concentration of leucine under phase contrast microscope. Primary preterm rat satellite cells reaching approximately 80% confluence are cultured in differentiation medium, with different concentrations of leucine for, three days, and then were observed under phase contrast microscope. (**A**) 0 mM leucine; (**B**) 0.1 mM leucine; (**C**) 0.5 mM leucine; (**D**) 2.0 mM leucine; (**E**) 2.0 mM leucine + 50 nM Rapamycin. (×200).

To initiate differentiation, primary satellite cells reached approximately 80% confluence were cultured in differentiation medium for 3 days. When cultured in the differentiation medium containing 0.5mM leucine, cell proliferation rates slowed down, and cells fused together to form myotubes. After induced by differentiation medium for 3 days, cells fused extensively to form thick myotubes (Figure 4C), most of which showed “rhythmic contraction” phenomenon. However, in the absence of leucine differentiation medium, cell differentiation was poor and only short myotubes were seen (Figure 4A). Furthermore, in differentiation medium containing 0.1 mM leucine, cell differentiation remains poor, only long, thin myotubes form without obvious “rhythmic contraction” phenomenon (Figure 4B). While increasing leucine concentration to 2.0 mM, cell differentiation improved. Both rough and pull net-like myotubes formed with obvious “rhythmic contraction” phenomenon (Figure 4D). However, with addition of 50 mM rapamycin, cell differentiation was inhibited and “rhythmic contraction” phenomenon is not seen (Figure 4E).

### 3.4. Involvement of mTORC1 in Leucine-Stimulated Differentiation of Primary Satellite Cells

**Figure 5:**
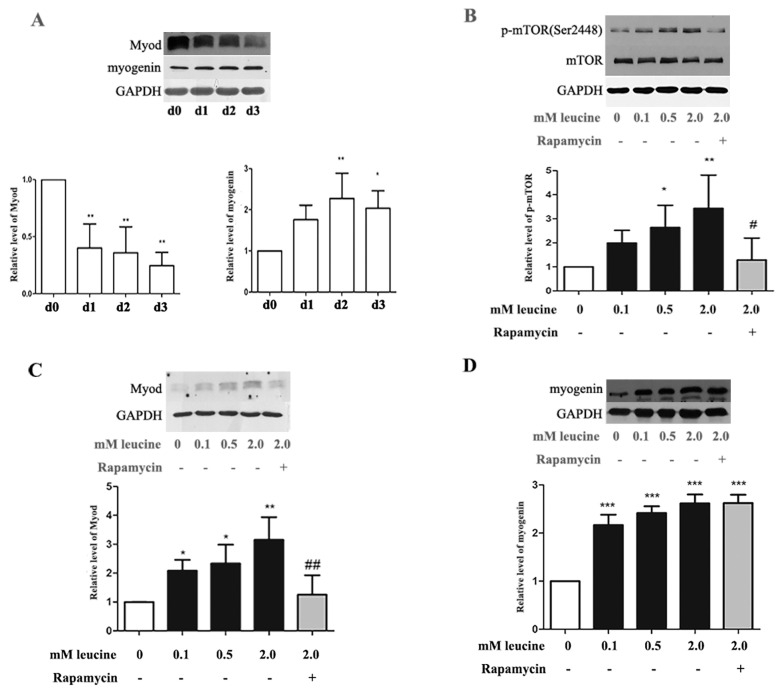
Involvement of mTORC1 in leucine-stimulated differentiation of primary satellite cells. (**A**) Primary preterm rat satellite cells reaching approximately 80% confluence, were induced to differentiate by differentiation medium. Cells lyse every 24 h and the lysates subjected to Western analysis. MyoD and myogenin densitometry values were adjusted to GAPDH intensity, and then normalized to the control group (d0). * *p* < 0.05, ** *p* < 0.01 vs. control; (**B**) Confluent primary satellite cells cultured in differentiation medium with varying concentrations of leucine for 1 h. When indicated by “+”, cells received 50 nM rapamycin. We used western blot assay to detect the expression of mTOR and phospho-mTOR. Phospho-mTOR densitometry values were adjusted to total mTOR intensity, and then normalized to expression from the control group (0 mM leucine); (**C**) Confluent primary satellite cells were cultured in differentiation medium with different concentrations of leucine for 8 h, followed by western blot analysis. MyoD densitometry values were adjusted to GAPDH intensity and then normalized to expression from the control group (0 mM leucine); (**D**) Confluent primary satellite cells were cultured in differentiation medium with different concentrations of leucine for 3 days, followed by Western analysis. Myogenin densitometry values were adjusted to GAPDH intensity and then normalized to expression from the control group (0 mM leucine). * *p* < 0.05, ** *p* < 0.01, *** *p* < 0.01 vs. control (0 mM leucine). ^#^ *p* < 0.05, ^##^ *p* < 0.01 vs. 2.0 mM leucine. All data are shown as the mean ± SD of three independent experiments and representative images are shown.

In the differentiation progress of primary preterm rat satellite cells, the expression of MyoD decreased while myogenin increased gradually (Figure 5A). Leucine activated phosphorylation of mTOR in a dose-dependent manner in differentiating cells (Figure 5B). In order to test whether or not leucine plays a functional role in differentiating primary satellite cells, the cells were subject to different concentrations of leucine and rapamycin for 8 h and 3 days respectively, then tested by western blot. The results showed that leucine promoted the protein expression of MyoD in the early differentiation and rapamycin decreased the expression of MyoD (Figure 5C). Furthermore, leucine increased the level of myogenin in the late differentiation of myotubes. However, the protein level of myogenin was not affected by rapamycin treatment (Figure 5D). 

## 4. Discussion

In this study, we aimed to investigate the role of leucine in the process of the proliferation and differentiation of primary preterm rat satellite cells, and the relationship with mTORC1 signal pathway. To the best of our knowledge, our study successfully established a primary preterm rat satellite cell culture system *in vitro* which was not been previously reported. We showed that leucine might up-regulate the activation of mTORC1 to promote proliferation and differentiation of primary preterm rat satellite cells. We also demonstrated that leucine promoted the differentiation of myotube in part through mTORC1–myoD signal pathway.

The gestation period of a rat is generally 21 to 22 days. In the lung disease research, preterm rats were born by caesarean section on day 19 or 20 of gestation [27,28]. Our study selected the day 18 of gestation to deliver preterm rat as the preterm animal.

The method of primary satellite cell isolation mainly includes mechanical shearing and enzymatic digestion, releasing the cell from the muscle cell membrane and basement membrane [29]. We obtained primary preterm rat satellite cells by digestion with type I collagenase and trypsin, and purification via pre-plating, which could allow reaching purity around 90%–95% of satellite cells. Desmin is one of the myogenic marker proteins expressed in the early differentiation of muscle satellite cells. These proteins are missing in non-myogenic cells. Owe to its significance and characteristics, desmin is a specific marker for skeletal muscle cells [29]. Primary muscle satellite cells have an essential biological characteristic in that it can differentiate into myotubes under special conditions, which is an important indicator to identify muscle satellite cells in morphology. Our study demonstrated that primary preterm rat satellite cells could fuse with each other and form myotubes under the differentiation medium containing 2% horse serum.

Leucine, one of the indispensable branched-chain amino acids, is an important regulator of muscle mass through the control of protein synthesis [30]. Recently, Averous, *et al.* found that leucine limitation prevents the differentiation of both mouse-derived C2C12 myoblasts and primary satellite cells [31]. Chen *et al.* demonstrated that leucine could promote proliferation of C2C12 myoblasts [32]. These findings suggest that leucine plays more biological functional roles beyond the fundamental role as a substrate for protein synthesis in skeletal muscle tissue. Here, we prove that leucine could promote proliferation of primary preterm rat satellite cells in a concentration dependent manner.

We know that mTOR and its downstream target S6K1 play an important role in the regulation of cell proliferation [33,34]. Varma *et al.* demonstrated that rapamycin leads to a decline in the HepG2 cell proliferation through the inhibition of mTOR [35]. In this study of primary preterm rat satellite cells, we found that leucine up-regulate the phosphorylation level of mTOR and S6K1 in a concentration dependent manner. Rapamycin down-regulated the phosphorylation level of mTOR and S6K1, and inhibited cell proliferation. Our observation illustrated that leucine may activate mTORC1 signaling pathways to promote proliferation of primary preterm rat satellite cells.

In the regulation of satellite cell differentiation, the role of leucine is still unclear. Our study designed differentiation medium containing different concentrations of leucine to induce the myotube formation of primary preterm rat satellite cells. We found that leucine had a differentiation-promoting effect on the formation of myotubes, and both MyoD and myogenin induced by leucine. These two lines of evidence indicate that leucine-induced up-regulation of MyoD and myogenin may contribute to leucine-induced differentiation promotion of primary preterm rat satellite cells.

Evidence now exists that mTOR is a master regulator of skeletal myogenesis, controlling multiple stages of the myofiber formation process [36]. However, the relationship between mTOR and MyoD is controversial. Indeed, studies show that rapamycin inhibited the differentiation of rat L6 myoblasts and mouse C2C12 cells [25,37]. However, neither S6K1 nor 4E-BP1 is the relevant effector for the myogenic signaling of mTOR [25]. These findings suggest existence of other downstream effectors of mTORC1 signaling pathways regulating myogenesis. Moreover, Sun *et al.* demonstrated that rapamycin treatment led to down-regulation of MyoD protein levels in C2C12 cells, which identify an mTOR-MyoD pathway that controls myocyte fusion during myoblast differentiation [26]. However, Averous *et al.* showed that rapamycin did not affect MyoD expression while leucine limitation decreased MyoD protein levels in C2C12 cells. Hence, they presumed that mTORC1 complex activity is not involved in the regulation of MyoD in response to leucine starvation [31]. We found that, in the early differentiation stage of primary preterm rat satellite cells, both the phosphorylation level of mTOR and the expression of MyoD induced by leucine and rapamycin treatment caused a decline of MyoD protein levels. It is conceivable that leucine increases the phosphorylation of mTOR and subsequently up-regulates the protein level of MyoD, which governs myocyte fusion in the myogenesis process. Our results suggested that a leucine-mTORC1-MyoD signaling pathway plays an important regulatory role during differentiation of primary preterm rat satellite cells, which is consistent with the finding of Sun *et al.* [26]. 

In the later differentiation stage of primary preterm rat satellite cells, leucine also up regulated the protein level of myogenin, but rapamycin did not decrease its expression. We can conclude that mTOR is not involved in the regulation of myogenin in response to leucine. Therefore, the mechanism that leucine regulates in myogenesis is not limited to the mTOR signal pathway. It remains necessary to identify the signaling pathway and the molecular mechanism involved in the regulation of myogenesis by leucine. 

## 5. Conclusions

We show that leucine might up-regulate the activation of mTORC1 to promote proliferation and differentiation of primary preterm rat satellite cells. We also demonstrate that during the process of myotube formation, leucine promotes differentiation in part through mTORC1-MyoD signal pathway. However, mTOR was not involved in the regulation of myogenin. Further research needs to illustrate the mechanism involved in the regulation of myogenesis by leucine. 
