# Correction: Dai et al. Leucine Promotes Proliferation and Differentiation of Primary Preterm Rat Satellite Cells in Part through mTORC1 Signaling Pathway. *Nutrients* 2015, *7*, 3387–3400

**DOI:** 10.3390/nu17223554

**Published:** 2025-11-14

**Authors:** Jie-Min Dai, Mu-Xue Yu, Zhen-Yu Shen, Chu-Yi Guo, Si-Qi Zhuang, Xiao-Shan Qiu

**Affiliations:** Department of Pediatrics, The First Affiliated Hospital, Sun Yat-sen University, Guangzhou 510080, China; daijm6639@foxmail.com (J.-M.D.); shenzhy@mail.sysu.edu.cn (Z.-Y.S.); guochuyi1990@foxmail.com (C.-Y.G.); zhuangsq@mail.sysu.edu.cn (S.-Q.Z.); qiuxsh@mail.sysu.edu.cn (X.-S.Q.)

In the original publication [1], there was a mistake in Figure 5D. The Western blot image was mistakenly uploaded. The authors formally requested the replacement of the Western blot image presented in Figure 5D. The corrected Figure 5D appears below. The authors state that the scientific conclusions are unaffected. This correction was approved by the Academic Editor. The original publication has also been updated.

## Figures and Tables

**Figure 5:**
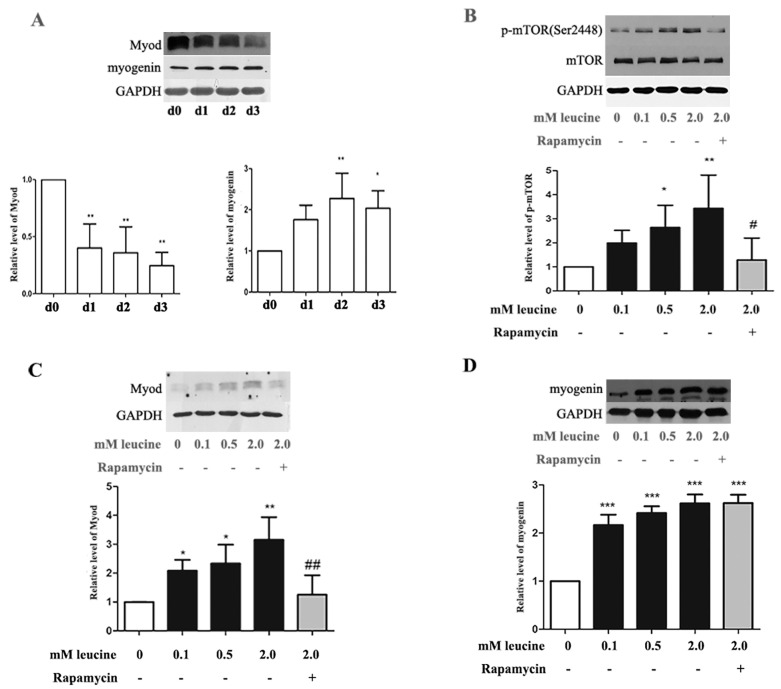
Involvement of mTORC1 in leucine-stimulated differentiation of primary satellite cells. (**A**) Primary preterm rat satellite cells reaching approximately 80% confluence, were induced to differentiate by differentiation medium. Cells lyse every 24 h and the lysates subjected to Western analysis. MyoD and myogenin densitometry values were adjusted to GAPDH intensity, and then normalized to the control group (d0). * *p* < 0.05, ** *p* < 0.01 vs. control; (**B**) Confluent primary satellite cells cultured in differentiation medium with varying concentrations of leucine for 1 h. When indicated by “+”, cells received 50 nM rapamycin. We used western blot assay to detect the expression of mTOR and phospho-mTOR. Phospho-mTOR densitometry values were adjusted to total mTOR intensity, and then normalized to expression from the control group (0 mM leucine); (**C**) Confluent primary satellite cells were cultured in differentiation medium with different concentrations of leucine for 8 h, followed by western blot analysis. MyoD densitometry values were adjusted to GAPDH intensity and then normalized to expression from the control group (0 mM leucine); (**D**) Confluent primary satellite cells were cultured in differentiation medium with different concentrations of leucine for 3 days, followed by Western analysis. Myogenin densitometry values were adjusted to GAPDH intensity and then normalized to expression from the control group (0 mM leucine). * *p* < 0.05, ** *p* < 0.01, *** *p* < 0.01 vs. control (0 mM leucine). ^#^ *p* < 0.05, ^##^ *p* < 0.01 vs. 2.0 mM leucine. All data are shown as the mean ± SD of three independent experiments and representative images are shown.
